# A154 CANCERS ASSOCIATED WITH INFLAMMATORY BOWEL DISEASE IN CANADA: A POPULATION-BASED ANALYSIS OF CASES AND MATCHED CONTROLS

**DOI:** 10.1093/jcag/gwac036.154

**Published:** 2023-03-07

**Authors:** S Coward, S K Murthy, H Singh, E I Benchimol, E Kuenzig, G Kaplan

**Affiliations:** 1 University of Calgary, Calgary; 2 The Ottawa Hospital, Ottawa; 3 University of Manitoba, Winnipeg; 4 The Hospital for Sick Children, Toronto, Canada

## Abstract

**Background:**

Individuals with inflammatory bowel disease (IBD) are known to have a higher risk of digestive tract cancers and cancers associated with immunosuppression. As the IBD population is ageing, age-related cancers may be more commonly diagnosed.

**Purpose:**

To assess whether IBD patients were at a higher odds of incident cancers than their matched controls stratified by age above and below 65 years.

**Method:**

A population-based surveillance study was conducted in Alberta, Canada (April 1, 2002 to March 30, 2018). A validated algorithm identified cases of IBD. Each case was age and sex matched to up to 10 non-IBD cases from the general population and linked to the Alberta provincial cancer registry to extract pathology-confirmed incident cancer. Controls were removed if they were not residents of Alberta at the time the matched case was diagnosed with IBD. Only incident cancers diagnosed after the diagnosis of IBD (or matched indexed date for controls) were considered. Age was calculated based on year of inclusion in the cohort or, if applicable, the year of cancer diagnosis. Cancer diagnoses were classified: bladder, biliary and liver, breast, cervix, colorectal, endometrium, gastrointestinal, gynecological, head and neck, hematological, kidney, lung, melanoma, neurological, non-melanoma, pancreas, prostate, renal and bladder, small intestine, thyroid, and miscellaneous. Odds ratios (OR), with 95% confidence intervals (CI), compared IBD cases to matched controls using conditional logistic regression. Stratified analysis at age 65 (<65 and ≥65) was done for all cancers.

**Result(s):**

Overall, 3695 incident cancers were diagnosed among 35,763 individuals with IBD as compared to 22,687 cancers among 289,212 controls (OR:1.12; 95%CI: 1.08, 1.16). Those less than 65 years old were at higher odds of developing cancer (1.20; 95%CI: 1.15, 1.26) than those ≥65 (0.97; 95%CI: 0.90, 1.04). Those with IBD had a higher odds biliary and liver (7.41; 95%CI: 5.58, 9.84) and gastrointestinal (2.26; 95%CI: 2.06, 2.48), which including: colorectal (1.78; 95%CI: 1.57, 2.02), pancreas (7.79; 95%CI: 5.53, 10.97), and small intestine (6.59; 95%CI: 4.65, 9.35). Melanoma and non-melanoma, head and neck, and thyroid cancers did not have an increased odds but hematological, lung, neurological, and kidney cancers did show an increased odds among those with IBD. Cancers outside of the gastrointestinal tract were at a lower odds for IBD patients, including: bladder (0.68; 95%CI: 0.54, 0.87), breast (0.72; 95%CI: 0.64, 0.81), gynecological (incl. cervix (0.68; 95%CI: 0.61, 0.78) and endometrium (0.48; 95%CI: 0.34, 0.66), and prostate (0.64; 95%CI: 0.57, 0.73).

**Image:**

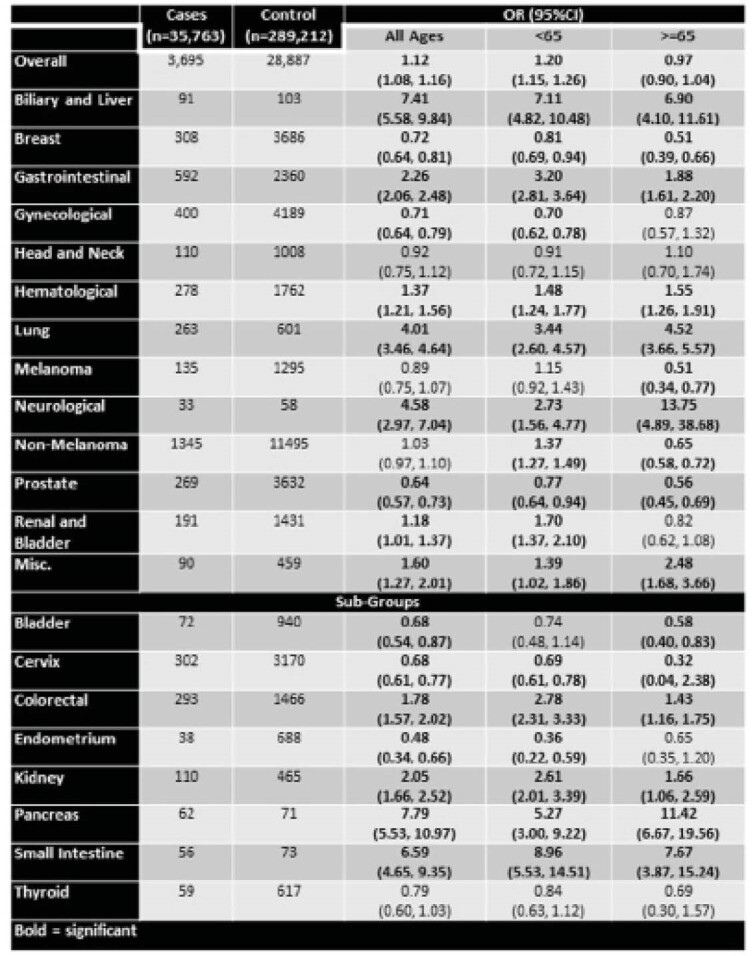

**Conclusion(s):**

Under the age of 65, individuals with IBD have a higher odds of being diagnosed with cancer than the general population, with cancers of the digestive tract driving this association across the age spectrum. Healthcare providers should be aware of higher occurrence of hematological, neurological, lung and renal cancers in those with IBD.

**Please acknowledge all funding agencies by checking the applicable boxes below:**

CIHR

**Disclosure of Interest:**

S. Coward: None Declared, S. Murthy: None Declared, H. Singh Consultant of: Pendopharm, Amgen Canada, Bristol Myers Squibb Canada, Roche Canada, Sandoz Canada, Takeda Canada, and Guardant Health, Inc., E. Benchimol Consultant of: Hoffman La-Roche Limited and Peabody & Arnold LLP for matters unrelated to medications used to treat inflammatory bowel disease and McKesson Canada and the Dairy Farmers of Ontario for matters unrelated to medications used to treat inflammatory bowel disease., E. Kuenzig: None Declared, G. Kaplan Grant / Research support from: Ferring, Janssen, AbbVie, GlaxoSmith Kline, Merck, and Shire, Consultant of: Gilead, Speakers bureau of: AbbVie, Janssen, Pfizer, Amgen, and Takeda

